# Five Factors of Frailty: A Multidimensional Psychometrically Robust Research Tool

**DOI:** 10.21203/rs.3.rs-8050875/v1

**Published:** 2026-07-06

**Authors:** Stacey Voll, Graciela Muniz-Terrera, Stuart W.S. MacDonald

**Affiliations:** University of Victoria; Ohio University; University of Victoria

**Keywords:** Frailty, Longitudinal, Psychometrics, Multidimensionality

## Abstract

**Background:**

Availability of large longitudinal population health and aging studies, such as the English Longitudinal Study on Ageing, hold many opportunities for the examination of the development of Frailty over time. Lacking are psychometrically robust longitudinal measurement of the concepts of frailty required for accurate and reliable estimates and identification of frail individuals.

**Objectives.:**

Development of a psychometrically robust longitudinal research tool of Frailty. The tool is to be easy to replicate for future use in health and aging research.

**Participants/Measurements.:**

Data comes from four waves of ELSA (2014–2017); *N*=39,528. Six domains of health status deficits that may measure frailty were used: Mobility, Daily Function, Self-rated general health, Self-rated pain, Depression, Self-report doctor diagnoses of health.

**Design.:**

Exploratory factor analysis (EFA) of common-self-reported deficits available across four waves of longitudinal data were compared to determine which deficits were a best fit for a research-based frailty index. Standardized regression factor scores were calculated to represent individual’s placement in factors identified from EFA. Cross-sectional validation and reliability are reported.

**Results.:**

Five-Factors of Frailty found were consistent across ELSA waves. Mobility/Severe Pain, Planning/Self Care, Depression, Moderate Pain/Movement/Arthritis and Cardio-Metabolic. The variable make-up of each factor differed between males and females. Psychometric properties of the five factor frailty model were taken into account at each stage of development (factorability/sampling adequacy, cross-sectional replicability, internal consistency, low interdeterminacy, relations with age).

**Conclusions.:**

Five distinct factors of an accumulation of deficits approach to frailty were found. Use and development of the five factors of frailty research tool are outlined. This research tool will aid researchers in determining the complex risk factors and outcomes of empirically derived components of frailty in ELSA.

## BACKGROUND

Frailty has become an increasingly important topic in the field of health research. Yet, there is no agreement on how to measure frailty or identify adults as frail, resulting in high heterogeneity between estimates of frailty and identification of frail individuals.^1,2^ The need to establish standard measures for this concept that is appropriate to the purpose, setting and population is required.^3^ The purpose of this study is to provide a frailty measure with robust psychometric properties to be used in research using longitudinal health study sets.

Overall, there are two broad research approaches to the construct of frailty: Frailty as the accumulation of deficits across different health domains^4^ and a phenotype model of frailty as a decline in physical functioning^5^. Their characteristics, uses, advantages and limitations have been extensively discussed in the literature before.^6–14^ The first listed approach is taken into consideration. Searle et al.^10^ suggest that health status deficits should relate to an increase in prevalence with age, but not saturate too early in aging (eg. before the age of 50) and should cover a broad range of health systems. This lends easily to the creation of a Frailty Index by calculating the sum of deficits in the individual divided by the number of potential deficits. Our concern of this valuable approach is the use of as many deficits available to a researcher, with availability to large longitudinal data sets, without verification in how such deficits play a role in, or accurately measure, the concept of frailty.

We used the accumulation of deficits theoretical approach to frailty developed by K. Rockwood and A. Mitnitski in 2007^4^. Within this approach, the deficits used should cover a broad range of health systems. Our concern of this approach is the use of as many health deficits available to a researcher without understanding how deficits play a role in the measurement of frailty. For example, Searle et al^10^ suggest building a Frailty Index by calculating the sum of deficits in the individual divided by the number of potential deficits. This is a method that has aided many health researchers to be able to use data from large population health longitudinal studies available. Standard operating procedure require a minimum of 30 deficits, associated with health status, present in each wave of data, from several different health systems^10^. Our concern of this approach is the use of as many deficits available to a researcher without verification that such deficits play a role in, or accurately measure, the concept of frailty. As well, using non-refined sum of scores (as a cut-off, standardized variable, or a weighted score) is a good method when building a self-report clinical tool, with a construct that could provide external validation. But here we are attempting to build a tool of measurement for a construct that does not have clear external validation standards, a challenge outlined over five years ago in Sutton et al.’s 2016^11^ systematic review of the psychometric properties of frailty tools.

### OBJECTIVES

The purpose of this study is to determine which deficits are the best fit for a longitudinal research measure specific to ELSA, using the accumulation of deficits concept of frailty. Exploratory and confirmatory sets of factor analyses are compared, using four waves of longitudinal health data to ensure stability among deficit loadings and dimensions.

### PARTICIPANTS

Data were derived from the English Longitudinal Study of Aging (ELSA).^15^ ELSA is a representative sample of community-dwelling respondents aged 50 or older in England. Data collection for ELSA started in 2002, with follow-up data collection every two years. This study used four waves of data collected in 2004–05 (wave 2, v4 N=9,432), 2008–09 (wave 4: a respondent refreshment, v3 N=11,050), 2012–13 (wave 6: a respondent refreshment, v2 N=10,601) and 2016–17 (wave 8, v2 N=8,445) of the ELSA core data. The ELSA data received ethical approval from the appropriate institution at each wave (wave2 MREC/04/2/006; wave4 07/H0716/48; wave6 11/SC/0374; wave8 15/SC/0526). Approval information for each wave can be found at: https://www.elsa-project.ac.uk/ethical-approval. Respondents with non-zero-weights were used at each wave, resulting in final samples of participants 52–85 years old (wave 2 n=8,780, wave 4 n=9,805, wave 6 n= 9,608 and wave 8 n = 7,133).

### MEASUREMENTS

Self-reported variables, from ELSA Respondents, available across nine modules of variables were use: Activities of Daily Living (ADL), Instrumental Activities of Daily Living (IADL), Mobility, General Health, Fallen, Incontinence, Pain, Depression via CESD-8, and Chronic Conditions (weighted percentages reported in the Appendix). Overall, 53 self-reported health variables were examined. All deficits were coded as 1 if present and 0 if absent.

## DESIGN

To first determine the best number of dimensions and the consequential variables for each wave, preliminary Exploratory Factor Analysis (EFA) models with no rotation were run. Criteria for the number of dimensions extracted for each wave of data were based the strongest saturation point of the total variance accounted for (scree plots). Variables not meeting criteria (loading eigenvalues <0.200 on Principal Component Analysis Extractions) were removed from the preliminary EFAs. Once a consistent set of variables were determined among the four sets of ELSA waves, a refinement of the frailty measure was made. Comparisons of the non-rotated, Oblimin, and Varimax rotations of EFA models were used to determine the variance of factor loadings, unambiguous variables and the best fitting model. Kaiser Meyer Olkin (KMO) were used to measure the sampling adequacy, with values of 0.500 as a minimum criterion for EFA model acceptance. Bartlett’s test were used to indicate whether variables were correlated (p<0.001). Examination of original values explaining an underlying factor was made with criteria of communalities of <0.300 via component matrix of frailty factors for all waves.^16^ Standardized Regression factor scores from the EFA were used to predict the location of each individual on the factor. This procedure differs from the more commonly used sum-of-deficit method, as the sum score reflects the extent to which the broad component estimated is manifested by each individual case, while the regression factor score method uses an underlying model to predict factor scores. Using standardized regression scores, that have no limits (they can be negative or positive), the age the factor appears can be determined (when standardized regression scores pass the mean of 0). Also, the age the scores pass a diagnostic threshold (typically, and as used here, when standardized regression scores pass 0.20 – 0.25, aligning with the 95^th^ PR.^17^ By using specific cut-off scores at the 95^th^ PR, we are able to identify individuals of passing an empirically-derived threshold of diagnostic criteria of each factor of Frailty.

As first step in the analyses, potential differences between sex were examined, in response to findings of differing effects over time of sex and frailty.^18–20^ At each wave, there were significantly different associations between males and females in the components of developing a research measure for frailty. All analysis were run again with male and female samples separately. A summary table of these regression models are in Appendix Table A. To further justify building separate measures for females and males, equivalency tests^21^ for final factors are reported in Appendix Table A Internal Consistency for each factor composition was examined using Cronbach’s alpha. Items that decreased internal consistency were removed. To increase extrapolation of the exploratory factor analyses, results from Confirmatory Factor Analyses (R lavaan 0.6–15 package)^22^ are reported for the final five factor frailty measures.

## RESULTS

For the female sample, cancer and asthma did not meet criteria at each wave and removed from the refined EFA modelling of 51 variables (49 for wave 2). For the male sample, cancer, asthma and osteoporosis did not meet criteria and excluded from the refined EFA modelling of 50 variables. Five factors were determined to be best fit for all waves for both males and females using scree plots. Varimax Rotation was found to provide the best fit with loadings unique and most meaningful to groupings (eigenvalue loadings, KMO and Bartlett’s test statistics of rotation models found in Appendix Table B). These models were stable and replicable, at wave 2, wave 4, wave 6 and wave 8, suggesting low inter-determinacy. Summary descriptives of the standardized distribution of factor scores are reported in Appendix D).

The five factors that consistently emerged were difficulties with mobility and severe pain, difficulties in planning and self-care, depression, difficulties with movement, suffering with arthritis and moderate pain, and cardio-metabolic conditions.

### F1 MOBILITY/SEVERE PAIN.

**Figure 1:**
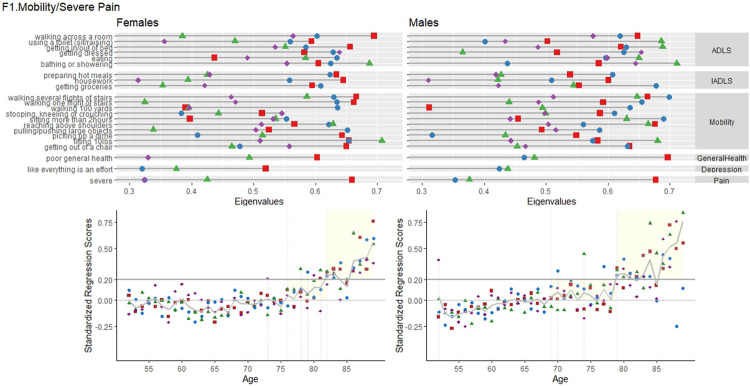
F1 Mobility/Severe Pain. Eigenvalue loadings (>.300) and Mean Standardized Regression Score across Age for four waves (red square=wave 2, blue circle=wave 4, green triangle=wave 6, purple diamond x=wave 8).

Factor 1, labelled “F1 Mobility/Severe Pain”, had consistent high loadings of the Mobility module, physical movement items of the IADL and ADL modules and self-reported poor general health and severe pain, for all waves. Figure 1 shows the variable loadings with eigenvalues <0.200 and the age of presentation for F1.

#### Females.

Variance accounted for this factor was similar across waves for females (12.02% (wave 2), 12.31% (wave 4), 13.93% (wave 6), 13.68% (wave 8)). Cronbach’s Alpha for all items with <0.200 eigenvalues were high ranging from 0.906 – 0.919 (23 to 25 items). Standardized Regression Mean Scores across age are visualized in Figure 1. F1 scores for females start to appear on average (passing scores of zero) at the age of 76 years and then crossing regression scores of 0.20 around 82 years old and onwards. Regression models significantly predicted the Standardized Regression Scores, based on age. For females, for every one year increase in age, there can be expected a 0.005 to 0.010 linear increase in F1 scores. Age accounted for 0.2% to 0.9% of the variance in F1 scores.

#### Males.

For all waves, variance accounted for the mobility factor was similar ((12.90% (wave 2), 13.62% (wave 4), 14.19% (wave 6), 13.78% (wave 8)). Cronbach’s Alpha for items with <.200 eigenvalues of the Mobility were high ranging from 0.907 −0.918 (22 to 29 items). Overall, Males had F1frailty issues appearing first around the age of 64, passing scores of 0.20, with increases after the age of 79 years. Significant linear increases of scores with age ranging from 0.010 to 0.015, with age accounted for 0.9% to 2% of the variance in F1 scores.

### F2 PLANNING/SELF-CARE

**Figure 2:**
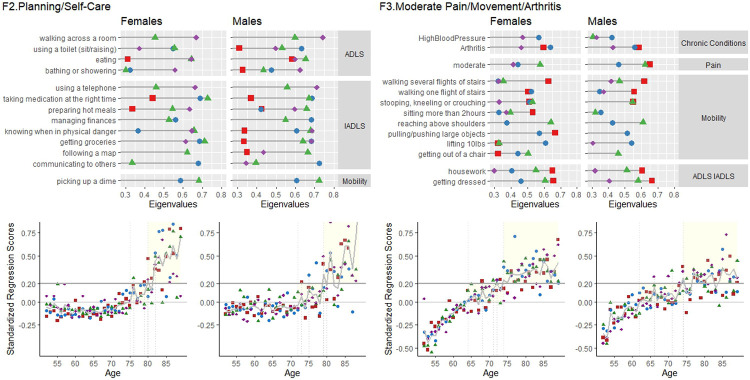
F2 Planning/Self-Care, F3 Moderate Pain/Movement/Arthritis. Eigenvalue loadings (>.300) and Mean Standardized Regression Score across Age for four waves (red square=wave 2, blue circle=wave 4, green triangle=wave 6, purple diamond x=wave 8).

Items involving organization, planning and self-care loaded consistently together. IADL items of having difficulties managing finances, following a map, taking medication on time, using a telephone and communicating had high loadings for both male and females across all four waves. The variable loadings, and age of presentation for F2 are shown in Figure 2.

#### Females.

For the female sample, this factor consisted of seven items with high loadings of the IADL module involving organization and memory, self-reported poor eyesight and hearing, having had a stroke and one to four ADL items. For the female sample (5.33% (wave 2), 6.13% (wave 4), 7.12% (wave 6), 6.37% (wave 8)). Cronbach’s Alpha for the items of F2 were high, ranging from 0.711 – 0.826 (9 to 15 items). F2 scores for females start to appear on average (crossing standardized regression scores of zero) at the age of 76 years and then crossing 0.20 around 82 years old and onwards (Figure 4). Regression models were significant in predicting the standardized regression scores based on age. Across all waves, for every one year increase in age, there can be expected a 0.009 to 0.018 linear increase in F2 scores. Age accounted for 0.8% to 3.2% of the variance in F2 scores.

#### Males.

For the male sample, this factor consisted of twelve items with high loadings. Along with high loadings of the IADL module involving organization and memory, and having poor eyesight, there were also loadings on preparing a meal, eating, hearing and having had a stroke. Variance accounted for this factor was consistent across waves for the male sample (7.74% (wave 2), 8.58% (wave 4), 7.65% (wave 6), 7.81% (wave 8)). Cronbach’s Alpha were high, ranging from 0.848 – 0.878 (13 to 21 items) for the males. F2 scores start to appear on average (crossing standardized regression scores of zero) at the age of 75 years and then crossing 0.20 around 84 years old and onwards (Figure 4). Significant linear increases of scores with age ranging from 0.011 to 0.013, with age accounting for 1.1% to 2.1% of the variance in F2 scores.

### F3 MODERATE PAIN/MOVEMENT/ARTHRITIS

Factor 3, labelled “F3 Moderate Pain/Movement/Arthritis”, had high loadings of living in moderate pain, having difficulties in gross motor-movements, such as stooping, kneeling, crouching, getting out of a chair, taking flights of stairs, and having arthritis. Differences between the female and male loadings for this factor were in getting dressed and strength (lifting 10 lbs, pulling/pushing large items) (Figure 2).

#### Females.

Variance accounted for in this factor of were slightly different across waves (7.49% (wave 2), 6.03% (wave 4), 5.43% (wave 6), 6.45% (wave 8), with high Cronbach’s Alpha high, ranging from 0.840 – 0.879 (14–17 items). For females, F3 scores appear around the age of 63–65, and crosses over the 0.20 threshold around the age of 64 to 73. Regression models were significant in predicting the Standardized Regression Scores of the F3 factor, based on age. Across all waves, for every one year increase in age, there can be expected a 0.021 to 0.027 linear increase in F3 scores. Age accounted for 4.4% to 7.0% of the variance in F3 scores.

#### Males.

**Figure 3:**
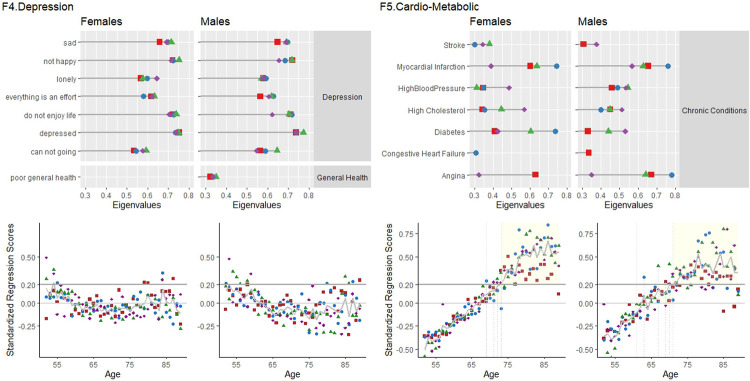
F4 Depression, F5 Cardio-Metabolic Conditions. Eigenvalue loadings (>.300) and Mean Standardized Regression Score across Age for four waves (red square=wave 2, blue circle=wave 4, green triangle=wave 6, purple diamond x=wave 8).

For the males sample, variance accounted for were slightly different across the four waves (6.33% (wave 2), 4.52% (wave 4), 5.64% (wave 6), 6.34% (wave 8)). Cronbach’s Alpha was high (.753 - .881 (11–18 items)). As seen in Figure 3, factor scores emerge around the ages of 59 to 64, and start to cross over the 0.20 threshold at 66 years old at wave 8, 71 at wave 6, 73 at wave 2 and 85 years old at wave 4. There were significant linear increases of scores with age, ranging from 0.01 to 0.02, with age accounting for 1.7% (Wave 2) to 5% (wave 8) of the variance in F3 scores.

### F4 DEPRESSION

F4 Depression had high loadings, for the female and male samples, of the Depressive Symptoms module (as measured by the CESD-8), poor general health and having ever been diagnosed with any emotional, nervous or psychiatric problem. The highest loadings were of feeling depressed, not happy, not enjoying life, feeling sad and feeling like everything was an effort (Figure 3).

#### Females.

Variance accounted for this factor was similar across waves ((7.30% (wave 2), 7.15% (wave 4), 7.65% (wave 6), 7.56% (wave 8)). For females, the factor of depression at waves 4, 6 and 8 emerged as the second factor, and for wave 2, as the third. Cronbach’s Alpha for the items of the F4 factor were high, ranging from 0.783 – 0.811 (10 items). Linear decreases in F4 scores with age were significant at waves 6 and 8, but not at waves 2 and 4. F4 scores for females appear around 50–59 years old, followed by a decrease at mid-age (60 to 70) and then another increase at older ages (79 and older).

#### Males.

Similar to the female sample, variance accounted for in this factor was 7% across all waves ((7.42% (wave 2), 7.48% (wave 4), 7.88% (wave 6), 7.49% (wave 8)). For males, the depression factor emerged as the third factor for waves 2, 4 and 8, and for as the second factor for wave 6. Cronbach’s Alpha was high across all waves (0.785 – 0.813, 10–11 items). Significant linear decreases of F4 scores with age were found for waves 6 and 8, but not wave 2 or 4. F4 scores are high for those 52 to 62 years old, low from 60 to 78 and then show a slight increase in later age, with variability between waves.

### F5 CARDIO-METABOLIC CONDITIONS

Factor 5, F5 Cardio-Metabolic Conditions, had high loadings of chronic conditions of angina, hypertension, diabetes, myocardial infarction, high cholesterol, congestive heart failure, and stroke (Figure 3).

#### Females.

The Cardio-Metabolic Conditions factor accounted for about 4% of the variance for the five-factor frailty measure, (4.51% (wave 2), 4.38% (wave 4), 4.36% (wave 6), 4.19% (wave 8). Cronbach’s Alpha for the items of the F5 Cardio-metabolic conditions factor ranged from 0.733 – 0.808 (15 – 18 items). Figure 3 show that for females, F5 scores appear around the age of 64 to 66, and cross over the 0.20 threshold around the age of 71 to 74. Regression models of F5 scores based on age were significant. Across all waves, for every one year increase in age, there was a 0.027 to 0.037 linear increase in F5 scores. Age accounted for 7.2% to 34.8% of the variance in F5 scores.

#### Males.

F5 accounted for 4% of the variance for the five-factor frailty measure (4.12% (wave 2), 4.18% (wave 4), 4.36% (wave 6), 4.00% (wave 8). Cronbach’s alpha was moderate to high ranging from 0.532 – 0.702 (8–12 items). Factor scores emerge around the ages of 61 to 64, and start to cross over the 0.20 threshold around 63 to 70 years old (Figure 3). Regression models showed significant linear increases of scores with age with a 0.022 to 0.035 increase with age accounting for 4.2% to 11.3% of the variance in F5 scores.

### USE OF THE FIVE FACTOR FRAILITY MEASURE

By using the eigenvalues as weights (Appendix Table C) an index score can be constructed for each of the five-factors of frailty.


$$\text{INDEX}\;\text{SCORE}=\left({\text{Variable}}_{1}*{\text{wave}\;\text{specific}\;\text{variable}}_{1}\text{eigenvalue}\right)+\left({\text{Variable}}_{2}*{\text{wave}\;\text{specific}\;\text{variable}}_{2}\text{eigenvalue}\right)+\ldots .\left({\text{Variable}}_{k}*{\text{wave}\;\text{specific}\;\text{variable}}_{k}\text{eigenvalue}\right)$$

Reduction of the number of variables can be made using the highest eignevalues for each factor; for instance, eignevalues of <0.300. For these reduced, wave specific, five-factor frailty measures, internal consistency and unidimensionality were examined. Cronbach’s alpha showed high levels of internal consistency for F1, F2, F3 and F4 (α<0.700) and medium levels for F5 (α<0.400). Variability accounted for each of the wave specific frailty factors suggest unidimensionality between the variables. To ensure internal validity of these reduced five factor frailty measures, Confirmatory Factor Analysis (CFA) models were run. CFA goodness of fit indices showed good fit. For Females at wave 8 and wave 4 (consecutively), Comparative Fit Index (*CFI*) values were 0.861 and 0.867; Tucker-Lewis (*TLI*) values were 0.850 and 0.857; Root Mean Square Error of Approximation *(RMSEA)* was 0.049 and 0.048; Standardized Root Mean Square Residual (*SRMR) was* 0.048 and 0.047. For Males, at wave 8 and wave 4, *CFI* values were 0.868 and 0.894; *TLI* values were 0.858 and 0.885; *RMSEA were* 0.050 and 0.044; *SRMR* was 0.049 and 0.042. All of the estimate coefficients loadings were significant. All variances were positive. These measures show good/reasonable model fit for the five factors determined by the Exploratory Factor Analyses models.^23,24^

## CONCLUSIONS

Five-factors of frailty were found to be broadly similar across four waves of ELSA. Mobility was a factor consisting of ADLS and IADLS involving function loss due to difficulties in movement, and suffering with severe pain for males. Depression consisted entirely of the CESD items and having been diagnosed with a psychiatric disorder. The factor labelled Planning/Self-Care had high loadings of ADL and IADL items involving problem solving, remembering and the ability to care for oneself. Suffering with moderate pain, arthritis and ADLS/IADLS involving gross motor movements emerged as a factor that differed between males and females in terms of loss of strength.

**Figure 4:**
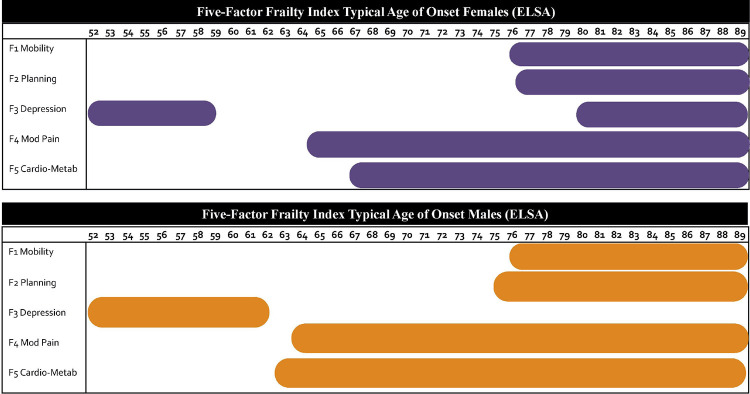
Five distinct factors of an accumulation of deficits approach to frailty were found: F1. Mobility/Severe Pain, F2. Planning/Self-Care, F3. Depression, F4. Moderate Pain/Movement/Arthritis and F5. Cardio-Metabolic. Standardized regression factor scores were calculated to determine individuals’ placement within the Five-Factors of Frailty. The typical pattern of the age of onset are unique for each frailty factor.

There were significant differences between females and males; across all waves, between 20–43% of females, and 18–38% of males were identified as experiencing onset with one of the five-factors of frailty (having frailty regression scores above zero) (Appendix Table D). Shown in the summary visualization of Figure 4, the age of onset differed among the factors of frailty for males and females. For females, moderate pain/arthritis and the cardio-metabolic conditions factors appear around age 63; for males, the age of onset is earlier for these factors, around the age of 59. Depression, for both females and males, appear in the late 50s to early 60s, decrease from 60s to 70s, and appear again around 70 years old. The onset of the mobility factor, overall, appeared around age 73 for females and age 70 for males. The Planning factor appeared around age 76 for females and 73 for males.

There has been substantial growth in the measurement and conceptualization of frailty within the literature. It is the intent of this paper to provide a research measure specific to ELSA data sets, to aid in quick and reliable research using the ELSA data sets. The factors determined across four waves, support familiar themes and results of frailty literature. For example, understanding a concept of frailty and functioning tasks that involve intrinsic capacity^25^ has been brought forward as an important aspect in investigations into lifelong health trajectories. Beyond the mobility, disabilities in IADLS and ADLS may be also linked to “intrinsic capacity” as a cluster for cognition^26^. The Planning/Self-Care frailty factor here supports future study of multi-dimensional aspects of cognition and frailty over time. Depressive symptomology are often used as a component, or having strong associations with, frailty.^10,27^ Determination directional effects of depression and frailty has not been complete clear.^28^ With this measure, intra- and inter- individual patterns of depression and defined factors of frailty can be examined over time. Arthritis Syndrome, a syndrome involving pain, measures of movement and arthritis^29^, has been supported as an identifier or marker for deficits accumulation measurements of frailty. The five-factor frailty measure will provide easy means to examine this syndrome over time. Cardio-Metabolic Syndrome is frequently presented in the literature as mechanism with, perhaps, bidirectional relations to frailty^30^. Having standardized regression scores for the ELSA population will allow further lifelong bidirectional pathways between frailty and multimorbidity among cardio-metabolic conditions.

There are limitations to the building of this research measure. The generalizability of the findings, the cross-sectional nature of the measure and an examination of cohort effects restrict results to the sample studied in the direct periods they provided self-report information. The findings are limited to English adults, 52 to 85 years of age, residing in the community, and able to provide informed consent. Greater in-depth examination of period shifts and cohort trends of the five frailty factors would be valuable to understanding lifelong trajectories in frailty, health and aging. This empirically derived longitudinal research tool provides means of identification of five-factors of frailty in females and males in England.

## Supplementary Material

Supplementary Files

This is a list of supplementary files associated with this preprint. Click to download.

- Appendix.docx

## Data Availability

The datasets analysed during the current study are publicly available upon request in the ELSA repository https://datacatalogue.ukdataservice.ac.uk/series/series/200011#access-data.
